# Exploring the antioxidant and protective effects of *Marsdenia thyrsiflora* Hook.f. leaf extract against carbon tetrachloride-induced hepatic damage in rat models

**DOI:** 10.3389/fphar.2024.1463922

**Published:** 2024-10-22

**Authors:** Sushmita Saha, Abdulrahman Alshammari, Norah A. Albekairi, Tasniya Nahiyan Zulfiquar, Md Salman Shakil, Kakoli Rani Mondal, Milton Kumar Kundu, Milon Mondal, Mohammad S. Mubarak

**Affiliations:** ^1^ Department of Pharmacy, Jahangirnagar University, Dhaka, Bangladesh; ^2^ Department of Pharmacology and Toxicology, College of Pharmacy, King Saud University, Riyadh, Saudi Arabia; ^3^ Department of Pharmacy, Bangabandhu Sheikh Mujibur Rahman Science and Technology University, Gopalganj, Bangladesh; ^4^ Department of Mathematics and Natural Sciences, Brac University, Dhaka, Bangladesh; ^5^ Pharmacy Discipline, Khulna University, Khulna, Bangladesh; ^6^ Department of Chemistry, Tennessee State University, Nashville, TN, United States; ^7^ Department of Chemistry, The University of Jordan, Amman, Jordan; ^8^ Department of Chemistry, Indiana University, Bloomington, IN, United States

**Keywords:** *Marsdenia thyrsiflora*, hepatoprotective effect, antioxidant properties, oxidative stress, CCl_4_-induced liver damage

## Abstract

**Introduction:**

Medicinal plants are vital to healthcare, yet many remain unexplored. *Marsdenia thyrsiflora* Hook.f., from Bangladesh’s Bhawal Forest, lacks research on its medicinal properties, especially its antioxidant capacities and protection against CCl_4_-induced liver toxicity. This study aims to evaluate the antioxidant properties of *M. thyrsiflora* leaf extract to determine its protective effects on rodents against CCl_4_-induced liver injury.

**Methods:**

After extraction, the total phenol, flavonoid content, and antioxidant capacity of the leaf extract were measured using established protocols. Free radical scavenging abilities were evaluated with 2,2′-diphenyl-1-picrylhydrazyl (DPPH), nitric oxide (NO) assays. Additionally, reducing power was assessed through cupric-reducing and ferric-reducing assays. Based on the OECD 420 recommendation, acute toxicity was tested on Swiss albino mice to establish an effective and safe dosage. For the hepatoprotective study, Sprague-Dawley rats were pre-treated with *M. thyrsiflora* leaf methanolic extract (MTLM) at 250 and 500 mg/kg body weight, and CCl_4_ was administered to induce liver damage. Serum hepatic enzyme levels (alanine aminotransferase (ALT), aspartate aminotransferase (AST), alkaline phosphatase (ALP), gamma-glutamyl transferase (GGT)), lipid profile (total cholesterol, triglycerides), total bilirubin, and markers of lipid peroxidation (Malondialdehyde (MDA)) were measured. The activities of antioxidant enzymes, including superoxide dismutase (SOD), catalase (CAT), and glutathione (GSH) were also evaluated to assess oxidative stress.

**Results:**

The results demonstrated that MTLM, rich in phenolic and flavonoid content, exhibits significant antioxidant activities in DPPH and NO radical scavenging assays, as well as in reducing power assays. The acute toxicity study confirmed the safety of MTLM, with no adverse effects observed even at high doses. For the hepatoprotective study, rats were administered CCl_4_ to induce liver damage, followed by treatment with MTLM. Results showed that MTLM significantly reduces liver damage markers such as elevated serum hepatic enzyme levels, lipid profile, total bilirubin, and lipid peroxidation and improves the activities of GSH and key antioxidant enzymes such as SOD and CAT. Histopathological analysis corroborated these findings, displaying reduced necrosis, inflammation, and edema in liver tissues treated with MTLM.

**Conclusion:**

MTLM extract exhibits potent antioxidant and hepatoprotective properties. Its ability to attenuate oxidative stress, enhance antioxidant enzyme activities, and facilitate histopathological changes in the liver highlights its potential as a natural therapeutic agent for liver damage. However, further investigation is required to understand its molecular processes, safety profiles, and active component characterization.

## 1 Introduction

The liver, a multifaceted wonder in the human body, stands guard over our internal harmony. It acts as a tireless janitor, filtering and neutralizing internal and external toxins, playing a critical role in our health and wellbeing (Bogdanos et al., 2013). However, despite its remarkable resilience, this vital organ faces constant exposure to threats, primarily from medication, excessive alcohol intake, and exposure to environmental toxins or xenobiotics (Gu and Manautou, 2012). These harmful substances cause substantial harm to the liver cells, usually by triggering oxidative stress and lipid peroxidation. This finally increases numerous biochemical indicators such as transaminases, bilirubin, and cholesterol, which ultimately lead to severe liver damage (Li et al., 2015). The lack of reliable and effective therapies for liver diseases makes their management a complex challenge (Panahi Kokhdan et al., 2017). This gap prompted interest in natural products, as their hepatoprotective compounds offer promising alternatives. Medicinal plants, boasting potent antioxidants and regenerative properties, can protect liver cells from damage and promote their repair, thus emerging as valuable associates in tackling this intricate health issue (Hong et al., 2015).

Understanding the intricate relationship between toxicant exposure and liver damage is crucial for developing effective treatments for a multitude of liver diseases. This quest for knowledge relies heavily on animal models, typically involving rodents, which are designed to replicate liver damage induced by toxins, drugs, or metabolic disturbances (McGill and Jaeschke, 2019). In this context, the carbon tetrachloride (CCl_4_)-induced liver damage model is well-recognized among diverse models (Zhai et al., 2023). This model is pivotal in understanding the mechanisms of liver damage, as CCl_4_ is metabolized into trichloromethyl and peroxyl trichloromethyl radicals by cytochrome P450 enzymes (Mondal et al., 2023). These radicals are known to initiate lipid peroxidation, leading to significant liver injury. This has drawn considerable attention to the role of antioxidants in protecting the liver from such oxidative damage, suggesting that they could potentially reduce the risk of liver diseases. In this regard, natural antioxidants have become a primary focus of research. There is accumulating evidence that medicinal plants, rich in antioxidant compounds, exhibit potent hepatoprotective activities (Meng et al., 2020). These plants improve the body’s antioxidant status, thus aiding in liver protection. The *Marsdenia* genus, belonging to the Apocynaceae family, is a notable example in this regard. Various species within this genus are known for their antioxidant, anti-microbial, anti-proliferative, anti-diabetic, and anti-inflammatory properties (Jiao et al., 2018; Wang et al., 2018).

*Marsdenia thyrsiflora* Hook.f. is a tropical climbing plant found in regions like Bangladesh, India, Malaysia, and East Asian countries (Rahman, 1990). In Bangladesh, it is often observed in the Bhawal Forest area of Gazipur; this vine-like plant thrives by intertwining with other flora. This plant has been traditionally used to treat various ailments, such as fever and stomachaches. Despite its potential, there is a scarcity of literature exploring its antioxidant and protective effects, sparking curiosity among researchers. Although tropical plants have been the source of many essential pharmaceuticals, research into their therapeutic properties has only touched on a small portion of the world’s plant species (Mendelsohn and Balick, 1995). This reality suggests that a vast, largely unexplored library of natural remedies exists, offering untapped potential for medical advancements. Responsible and sustainable exploration of these forest-derived plants could lead to the discovery of new, valuable pharmaceutical compounds. Thus far, no prior studies have explored the liver-protective or antioxidant properties of *M. thyrsiflora*’s leaf extracts. Therefore, this study aimed to evaluate these activities by testing the extract’s ability to protect rats from CCl_4_-induced liver damage.

## 2 Materials and methods

### 2.1 Chemicals and reagents

Chemicals and reagents used throughout this work were obtained from commercial sources and used as received. The following chemicals and reagents were purchased from Merck, Germany, and used without further purification: Folin-Ciocalteu reagent, sodium carbonate, gallic acid, aluminum chloride, potassium acetate, quercetin, sodium nitroprusside, and Griess reagent. Other reagents such as copper chloride, ammonium acetate, neocuproine, potassium ferricyanide, trichloroacetic acid, ferric chloride, ascorbic acid, DPPH (2,2-Diphenyl-1-picrylhydrazyl), sodium dodecyl sulfate, acetic acid, thiobarbituric acid, butanol, carbon tetrachloride, and pyridine were obtained from Sigma-Aldrich, United States.

### 2.2 Collection, identification, and extraction of plant material

Fresh leaves from *M. thyrsiflora* were collected from Bhawal Forest in Gazipur, Bangladesh in January 2018. A principal experimental officer from the Department of Botany at Jahangirnagar University identified and authenticated the plant. The leaves were rinsed, sun-dried, and then oven-dried at 40°C for optimal grinding conditions. They were then finely ground using a Jaipan blender. Approximately 1,000 g of this powdered plant material was subjected to Soxhlet extraction using 5,000 mL each of methanol and ethyl acetate at 65°C. The solution was then filtered using Whatman No. 1 filter paper and the extract was concentrated under reduced pressure using rotary evaporation at around 40°C. The resulting viscous extract was stored at −20°C for later examination.

### 2.3 Total phenol and flavonoid levels and total antioxidant capacity assays

The detection and determination of leaf extracts’ total phenolic, flavonoid content, and total antioxidant capacity were performed using established procedures. Determination of the phenolic compounds in the extracts was achieved using the method outlined by Hossain and colleagues. Briefly, 1.0 mL of the extract or various standard solutions were combined with Folin-Ciocalteu reagent and sodium carbonate, incubated, and then analyzed at 765 nm using a Shimadzu UV PC-1600 spectrophotometer (Hossain et al., 2017). Results of the phenolic content were expressed as gallic acid equivalents. For flavonoid content, the aluminum chloride colorimetric approach by Chandra et al. was employed (Chandra et al., 2014). In this method, a mixture of leaf extract, methanol, aluminum chloride, potassium acetate, and water was incubated at room temperature. The absorbance was then measured at 415 nm, expressing results in quercetin equivalents. Total antioxidant capacity was gauged using Wahed et al.'s phosphomolybdenum method (Wahed et al., 2019). This involved reacting leaf extract solutions with a reagent solution, heating, and measuring absorbance at 695 nm after cooling. The antioxidant activity was calculated and expressed as ascorbic acid equivalents.

### 2.4 DPPH and NO radical scavenging assays

The free radical scavenging abilities of the leaf extracts were assessed using two established methods. The DPPH assay evaluated their capacity to neutralize the stable free radical DPPH (Prieto et al., 1999). Extracts of ascorbic acid (the reference standard) at varying concentrations (6.25, 12.50, 25, 50, 100, 200, 400, and 800 μg/mL) were mixed with a DPPH solution and incubated in the dark for 30 min. Subsequently, the absorbance was measured, and the percentage of DPPH radical inhibition was calculated. Lower IC_50_ values indicated stronger DPPH scavenging activity. Additionally, the nitric oxide (NO) scavenging assay explored the extracts’ ability to neutralize the reactive NO free radical (Hossain et al., 2017). Extracts or a standard (6.25, 12.50, 25, 50, 100, 200, 400, and 800 μg/mL) were incubated with sodium nitroprusside to generate NO. After 2-hour incubation, the mixture reacted with Griess reagent, producing a colored product. Absorbance was again measured, and the percentage inhibition of NO was determined.

### 2.5 Reducing power capacity assays

In this study, the extracts’ ability to reduce copper ions and ferric cyanide was investigated, indicating their potential antioxidant effect. The cupric-reducing antioxidant capacity assay determines the extracts’ ability to reduce Cu^2+^ ions to Cu^+^ (Wahed et al., 2019). For this, extracts or a standard (concentration 6.25, 12.50, 25, 50, 100, 200, 400, and 800 μg/mL) were mixed with copper (II) chloride, ammonium acetate buffer, neocuproine, and water and incubated for an hour. Absorbance at 450 nm revealed the extent of copper reduction, with higher values indicating stronger capacity. The reducing power capacity assay also evaluates the extracts’ ability to reduce Fe^3+^ ions in potassium ferricyanide to Fe^2+^ (Wahed et al., 2019). Extracts and standards (concentration 6.25, 12.50, 25, 50, 100, and 200 μg/mL) were incubated with potassium ferricyanide at 50 °C, reacted with trichloroacetic acid, and centrifuged. The supernatant was further reacted with ferric chloride, and absorbance at 700 nm indicated the amount of reduced Fe^2+^ present. Higher absorbance reflected greater reducing power and potential antioxidant activity.

### 2.6 Experimental animals

For *in-vivo* studies, healthy male Sprague-Dawley rats (130–170 g) and male Swiss albino mice (25–30 g) were used throughout this investigation. These animals were housed in a controlled environment, with temperatures around 25°C ± 2°C, 50% ± 5% humidity, and alternating 12-hour light-dark cycles. Before the study, they had a week-long adaptation period with unrestricted access to food and water. Different animal models were chosen for acute oral toxicity and *in vivo* hepatoprotective studies to align with established protocols and maximize the reliability of the results. Swiss albino mice were used for the acute toxicity study due to their sensitivity to toxic substances and their widespread use in toxicity testing (Schlede et al., 1995). In contrast, Sprague-Dawley rats were selected for the hepatoprotective study due to their larger size, which facilitates blood and tissue sampling, and their well-documented response to CCl_4_-induced liver damage (Byun et al., 2018).

### 2.7 Acute toxicity study

A total of forty male Swiss albino mice, 8–10 weeks of age were randomly divided into four groups of ten mice each, using the OECD No. 420 standards for evaluating acute toxicity (OECD, 2002). Methanol leaf extracts of *M. thyrsiflora* were delivered orally at doses of 250, 500, 1,000, and 2,000 mg/kg body weight using a syringe with a stainless-steel needle, assuring targeted delivery to the stomach. Following the treatment, the mice were carefully observed for 24 h, with special attention given to the first 4 h to detect any indications of toxicity. Indicators included changes in behavior, respiratory and cardiovascular rhythms, motor activity, reflex reactions, and differences in skin or fur condition, and the duration of this observation period was 14 days.

### 2.8 Experimental design for hepatoprotective study

For the hepatoprotective study, male Sprague-Dawley rats were used due to their susceptibility to CCl_4_-induced liver toxicity, a factor well-supported by published reports (Khan et al., 2012; Masubuchi and Ihara, 2022; Siegers et al., 1984). The study’s dosing schedule was based on the CCl_4_ model using the method of Wong et al. with slight modifications (Wong et al., 2011). To induce liver damage, thirty-five rats were given intra-peritoneal injections of CCl_4_ mixed in olive oil (1:1) weekly, at a dose of 0.7 mL/kg body weight (BW). The rats were divided into five groups of seven animals each. The first group, the normal control group, received normal saline (0.9% sodium chloride). The CCL_4_ control group was given saline and CCl_4_. The silymarin group received silymarin (100 mg/kg BW) and CCl_4_. Two experimental groups labeled MTLM 250 and MTLM 500, were given methanol leaf extracts of *M. thyrsiflora* at 250 and 500 mg/kg BW, respectively, in addition to CCl_4_. The methanol leaf extracts were mixed with normal saline to achieve the required dosage and administered at a volume of 5 mL per group. All treatments were administered orally once daily at 9–10 a.m. for 28 days. At the end of the treatment period, the rats were humanely euthanized using a carbon dioxide (CO_2_) chamber, gradually introducing CO_2_ to ensure a painless death. Once unresponsive, blood was collected via cardiac puncture, and liver tissues were excised, weighed, and processed for biochemical analyses. Liver homogenates were prepared by homogenizing liver tissue in ice-cold phosphate-buffered saline (PBS) to achieve a 10% (w/v) homogenate. Specifically, 1 g of liver tissue was homogenized in 10 mL of PBS using a homogenizer (Rabrotech, India). The homogenate was then centrifuged at 10,000 × g for 15 min at 4°C, and the supernatant was collected for biochemical analyses, including antioxidant levels and lipid peroxidation assays. The relative liver weight, a marker of liver health, was also calculated as follows: wet liver weight/body weight × 100 (Wang et al., 2020).

### 2.9 Evaluation of biochemical indicators in the blood serum

This involved the evaluation of liver function through the measurement of several biochemical indicators. Key parameters analyzed were serum levels of enzymes and compounds indicative of liver health alanine and aspartate aminotransferases (ALT and AST), alkaline phosphatase (ALP), and lactate dehydrogenase (LDH) and Gamma-glutamyl transferase (GGT). Total bilirubin, proteins, albumin, globulins, triglycerides, total cholesterol, and cholesterol fractions (HDL-high-density lipoprotein and LDL-low-density lipoprotein) were also quantified. These assessments were conducted using standard procedures, utilizing human brand diagnostic kits with the protocol they provided. The analysis was carried out with the precision of a Humalyzer 3,500.

### 2.10 Evaluation of lipid peroxidation

For this, the malondialdehyde (MDA), a marker of lipid peroxidation, was measured in liver tissue using the thiobarbituric acid reactive substance assay (Ohkawa et al., 1979). Briefly, a small amount of liver homogenate (0.2 mL) was combined with a cocktail of chemicals, including sodium dodecyl sulfate, acetic acid, and thiobarbituric acid. This mixture was then heated at 95°C for an hour, cooled, and diluted before extraction with a butanol/pyridine mixture. The mixture was then centrifuged at 3,000 rpm for 10 min, and the extraction step separated the MDA into the organic layer, allowing its quantification through absorbance measurement at 532 nm. MDA levels were quantified and expressed as nanomoles per mg of protein.

### 2.11 Evaluation of reduced glutathione

A standard technique was used to detect reduced GSH. (Saidurrahman et al., 2022). After homogenizing the liver tissue, 4 mL of cold water was added, and 1 mL of 50% trichloroacetic acid was mixed in to determine the reduced GSH levels. The mixture was shaken intermittently for 10 min using a vortex mixer to ensure proper blending. The prepared mixture was then transferred to a centrifuge tube and centrifuged at 6,000 rpm for 15 min. The supernatant (2 mL) was collected and mixed with 4 mL of 0.4 M Tris buffer (pH 8.9). Subsequently, 0.1 mL of 0.01 M 5,5′-dithiobis-(2-nitrobenzoic acid) (DTNB) was added to the mixture. After allowing the reaction to proceed for 5 min, the absorbance was measured at 412 nm using a suitable blank, and the results were expressed as μg GSH/mg of protein.

### 2.12 Evaluation of antioxidant enzyme activities

To evaluate the effect of the *M. thyrsiflora* leaf extract on antioxidant defenses, the activity of two key enzymes, superoxide dismutase (SOD) and catalase (CAT), was measured. The SOD levels were determined by the method outlined by Nandi and Chatterjee (Nandi and Chatterjee, 1988). The procedure involved mixing tris-buffer and EDTA solution, after adding a small volume of the homogenate and varying amounts of pyrogallol. The solution’s absorbance was measured at 420 nm using a Shimadzu UV PC-1600 spectrophotometer; SOD levels were expressed as units per mg of protein. On the other hand, the CAT activity was quantified using a hydrogen peroxide-based method (Aebi, 1984). This assay included mixing homogenate with phosphate-buffered saline (PBS) in a cuvette and initiating the reaction with hydrogen peroxide. The absorbance was recorded at 240 nm with the aid of a Shimadzu spectrophotometer. The results for catalase activity were similarly expressed in units per mg of protein.

### 2.13 Histological analysis of hepatic tissue

After sacrificing the animals, livers were removed, sliced, and washed with saline. Tissues were then fixed in 10% neutral buffered formalin for histopathological examinations. Thin sections, each 6 μm thick, were then meticulously prepared from these samples embedded in paraffin wax, using an HM 325 rotary microtome from Thermo Scientific, U.K. Tissue sections underwent staining with hematoxylin and eosin to highlight their cellular structures. Finally, the stained sections were observed and captured using an Olympus DP 72 microscope (Tokyo, Japan) with images captured at 10X magnification (Mondal et al., 2021). The histological score analysis was done using a blinded method with three individuals. The evaluation was given as the total of the individual score grades of 0 (normal), 1 (mild change), 2 (mild to moderate severity), 3 (moderate severity), and 4 (serious severity) for each of the three parameters: necrosis, inflammation, and edema (Ren et al., 2019; Zhang et al., 2010).

### 2.14 Statistical analysis and data presentation

Results are expressed as the mean ± standard deviation (SD). Data were subjected to Student's t-test and one-way analysis of variance (ANOVA) with the aid of GraphPad Prism (version 6.02) (GraphPad Software, San Diego, California USA, http://www.graphpad.com) and Microsoft Excel 2013 software to assess the statistical significance. Furthermore, Dunnett’s and Tukey’s multiple comparison tests were employed for in-depth data examination. Statistical significance was determined at 5%, 1%, and 0.1% confidence levels, corresponding to *p* ≤ 0.05, 0.01, and 0.001, indicated as a, b, c, and x, y, z, respectively.

## 3 Results

### 3.1 Phytochemical contents

Table 1 presents a comparative analysis of the methanolic (MTLM) and ethyl acetate (MTLE) *M. thyrsiflora* leaf extracts, focusing on their total phenolic, flavonoid content, and overall antioxidant capacity. Results revealed that MTLM extract contains significant amounts of total phenols (217.25 ± 0.35 mg/g) and flavonoids (250.5 ± 3.54 mg/g) and exhibits remarkable antioxidant capacity (148.5 ± 3.54 mg/g), all expressed as the mean ± standard deviation (SD) from duplicate experiments. In contrast, the MTLE extract showed lower values: 59.75 ± 1.06 mg/g for phenols, 31.5 ± 2.12 mg/g for flavonoids, and 21.5 ± 2.12 mg/g for antioxidant capacity. The differences between the extracts were statistically significant (*p* < 0.05), as analyzed by Student's t-test.

**TABLE 1:** Total phenol, total flavonoid, and total antioxidant capacity of MTLM and MTLE.

Extracts	Total phenol (mg/g, GAE)	Total flavonoid (mg/g, QE)	Total antioxidant capacity (mg/g, AAE)
MTLM	217.25 ± 0.35^a^	250.5 ± 3.54^a^	148.5 ± 3.54^a^
MTLE	59.75 ± 1.06^b^	31.5 ± 2.12^b^	21.5 ± 2.12^b^

Data expressed as the mean ± SD, from two replicates. Distinct superscripts denote significant differences (*p* < 0.05) per Student’s t-test. MTLM, and MTLE, are the *M. thyrsiflora* methanolic and ethyl acetate leaf extracts, respectively.

### 3.2 Radical scavenging activities

**FIGURE 1:**
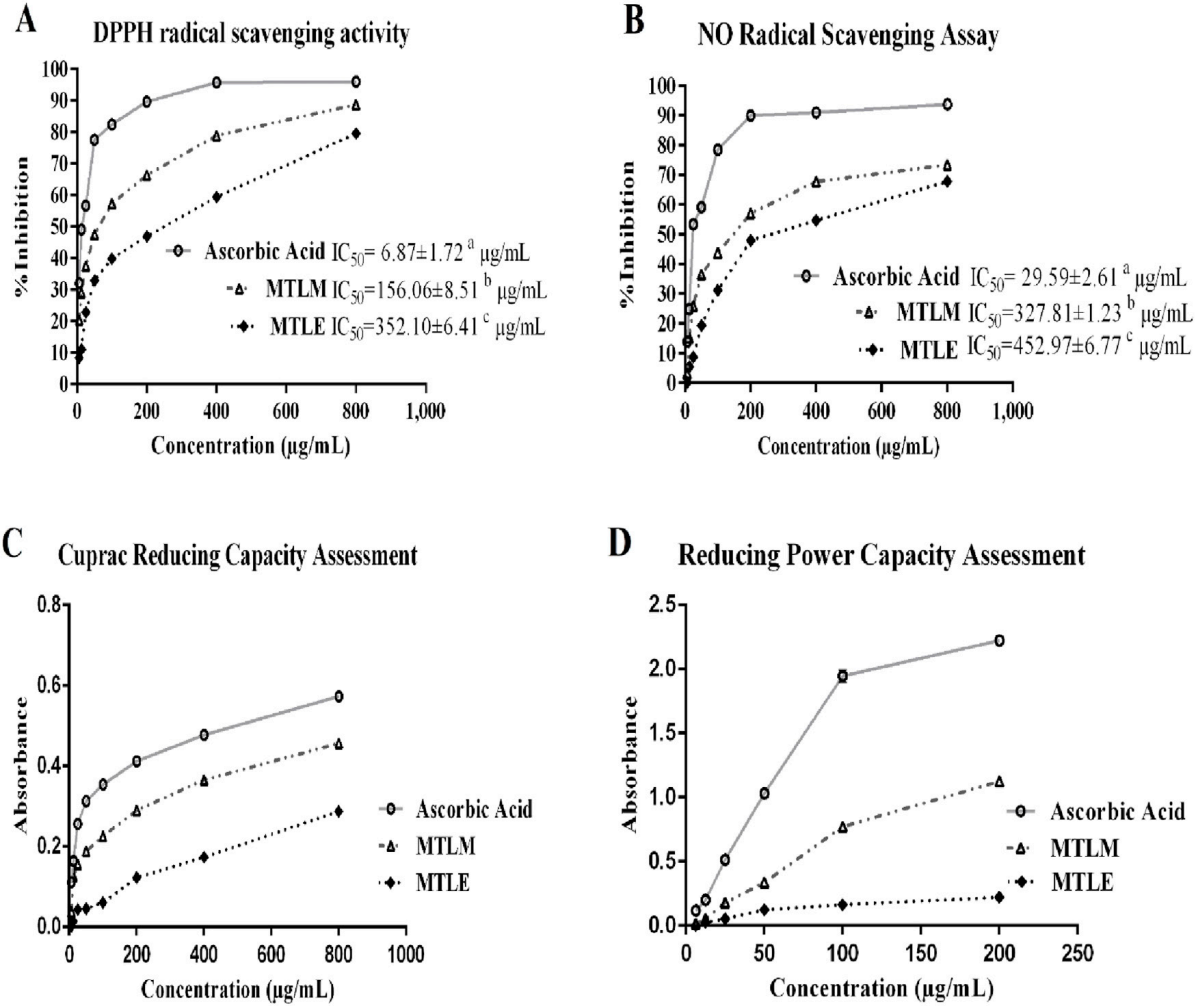
Radical scavenging and reductive antioxidant activities. **(A)** DPPH-free-radical-scavenging activity, **(B)** NO radical-scavenging activity **(C)** cupric reducing capacity, and **(D)** Ferric reducing power capacity of MTLM and MTLE and ascorbic acid. Data are analyzed using Tukey’s HSD and represents the mean ± SD from two replicates.

The DPPH and NO radical scavenging assays are benchmarks for the antioxidant efficacy of substances (Figures 1A, B). The MTLM and MTLE were pitted against ascorbic acid, a known antioxidant in these tests. Results showed that the MTLM extract demonstrates moderate scavenging activity, with its efficacy increasing as the concentration increases. On the other hand, MTLE’s activity was lower, indicating a less potent antioxidant effect. The half-maximal inhibitory concentration (IC_50_) values further illustrated this antioxidant strength. The assays underscored the potential of *M. thyrsiflora* extracts as antioxidants, with the methanolic form showing promising activity, albeit less than ascorbic acid, and the ethyl acetate form displaying a milder effect.

### 3.3 Reductive antioxidant activities

The reductive capacity of *M. thyrsiflora* in both extracts was determined through two essential antioxidant assays: the cupric and the reducing power capacity tests (Figures 1C, D). In the cupric assay, the MTLM and MTLE extracts demonstrated dose-dependent increases in absorbance, indicative of their electron-donating abilities, with MTLM exhibiting a more substantial capacity than MTLE. Similar results were obtained in the reducing power capacity assay, where the ability to reduce Fe^3+^ to Fe^2+^ increased with concentration. In this regard, ascorbic acid, used as a control, consistently showed the highest reductive potential. The increasing absorbance values with higher concentrations in both assays suggest that the methanolic extract has a substantial capacity to act as an antioxidant, with potential implications for its use in mitigating oxidative stress. Therefore, the methanolic extract was chosen for hepatoprotective activity assessment.

### 3.4 Acute toxicity study

In an acute toxicity study using Swiss albino mice, oral administration of the MTLM at a dose of 2000 mg/kg was well tolerated and did not result in any fatalities or adverse symptoms such as agitation, breathing difficulties, or neurological disturbances. Consequently, this dose level was identified as the Maximum Tolerated Dose (MTD). To explore the extract’s hepatoprotective properties, doses selected for the efficacy study were determined as fractions of the MTD. This approach is a standard practice in pharmacological research to ensure safety and efficacy. Accordingly, doses equivalent to 1/8th and 1/4th of the MTD, specifically 250 mg/kg and 500 mg/kg, were chosen for the hepatoprotective experiments. This strategy aims to balance the extract’s potential therapeutic effects with its safety profile, ensuring an effective yet non-toxic range for the in-depth analysis of its liver-protective capabilities.

### 3.5 Body and liver weight changes

Shown in Table 2 are the changes in body and liver weights of rats subjected to various treatments. Initial body weight (BW) was similar across all groups, indicating a balanced experimental setup. While all groups experienced weight gain, the CCl_4_ control group showed a significantly lower percentage of BW gain than the control, with a *p*-value of ≤0.05, suggesting a detrimental impact of CCl_4_ on overall growth. Relative liver weight, a marker of liver enlargement often associated with damage, was significantly higher in the CCl_4_ control group compared to the control (*p* ≤ 0.001), confirming liver injury. Interestingly, both doses of the *M. thyrsiflora* extract (MTLM 250 and MTLM 500) prevented this increase in relative liver weight, with values comparable to the control. Notably, the MTLM 500 group exhibited a relative liver weight nearly identical to the silymarin control, a well-established hepatoprotective agent (*p* ≤ 0.001). This suggests that a higher dose of *M. thyrsiflora* extract might offer protective benefits, such as silymarin.

**TABLE 2:** Effects of MTLM and CCL_4_ on body weight and relative liver weight.

Group	Initial BW (g)	Final BW (g)	BW gain (%)	Relative liver weight (%)
Normal Control	145.57 ± 9.29	165.43 ± 7.89	13.76 ± 2.71	3.87 ± 0.37
CCL_4_ Control	144.71 ± 9.34	157.71 ± 10.14	8.99 ± 1.09^a^	5.08 ± 0.41^c^
Silymarin Control	154.29 ± 9.11	172.14 ± 9.09^x^	11.61 ± 0.99	3.82 ± 0.17^z^
MTLM 250	151.57 ± 7.81	168.71 ± 7.41	11.37 ± 2.85	4.49 ± 0.19^a,y^
MTLM 500	150.14 ± 8.21	168.43 ± 7.30	12.26 ± 2.65	3.91 ± 0.28^z^

Results, with a sample size of 7, show varying significance: ‘a', ‘b', ‘c' for comparisons with the normal control, and ‘x', ‘y', and ‘z’ with the CCL_4_ control, determined by one-way ANOVA, as well as Dunnett’s and Tukey’s *post hoc* test.

### 3.6 Effects on hepatic enzyme profiles

**FIGURE 2:**
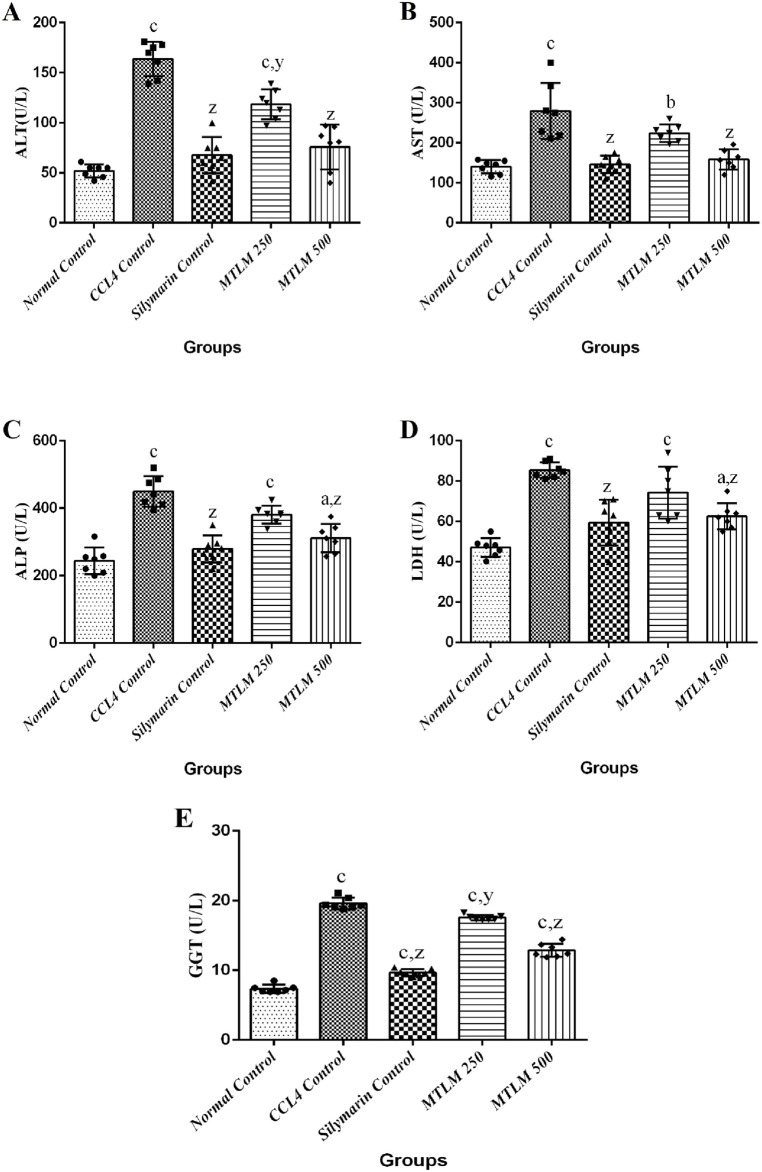
Effects of methanolic *M. thyrsiflora* leaf extract (MTLM) and CCL_4_ on liver enzyme profiles. **(A)** ALT, **(B)** AST, **(C)** ALP, and **(D)** LDH. Results, with a sample size of 7, show varying significance: ‘a', ‘b', ‘c' for comparisons with the normal control, and ‘x', ‘y', and ‘z’ with the CCL_4_ control, determined by one-way ANOVA as well as Dunnett’s and Tukey’s *post hoc* test.

The effects of MTLM extracts on liver enzyme levels were examined, providing insights into their hepatoprotective potential. Results showed that the level of ALT was significantly elevated in the CCl_4_ control group (*p* ≤ 0.001), signifying liver stress or damage. However, the groups treated with *M. thyrsiflora* extracts, particularly the higher dose of MTLM 500, showed reduced ALT levels (*p* ≤ 0.001), approaching the baseline established by the normal control group. Similarly, levels of AST were significantly increased in the CCl_4_ control group (*p* ≤ 0.001), confirming hepatic injury. Treatment with MTLM extracts mitigated this increase, with the MTLM 500 group demonstrating a notable decrease in AST (*p* ≤ 0.001), implying a restoration of hepatic health. In addition, levels of ALP followed this trend, with the CCl_4_ control group displaying significantly higher levels than the normal group (*p* ≤ 0.001). MTLM extracts, especially the 500 mg/kg dosage, effectively moderated the ALP increase (*p* ≤ 0.001), reflecting possible amelioration of bile duct obstruction or other hepatic alterations. Lactate dehydrogenase (LDH) levels, which reflect overall tissue damage, were also assessed. Results revealed that the CCl_4_ group exhibited a sharp increase (*p* ≤ 0.001), indicative of liver injury. In contrast, treatment with *M. thyrsiflora* extract, particularly the higher dosage, brought down LDH levels (*p* ≤ 0.001) compared to the negative control. Lastly, GGT, a sensitive biomarker for liver cell damage, particularly in the bile ducts, showed elevated levels in the CCl_4_ control group (*p* ≤ 0.001), indicating hepatic injury. The MTLM-treated groups (250 and 500 mg/kg) demonstrated dose-dependent reduction of GGT levels, with MTLM 500 showing a comparable effect (*p* ≤ 0.001) to Silymarin. These enzyme profiles suggest that *M. thyrsiflora* leaf extracts, especially at higher doses, have a potential hepatoprotective effect against CCL_4_-induced hepatic damage, and data are presented in Figure 2.

### 3.7 Effects on serum lipid profile

**FIGURE 3:**
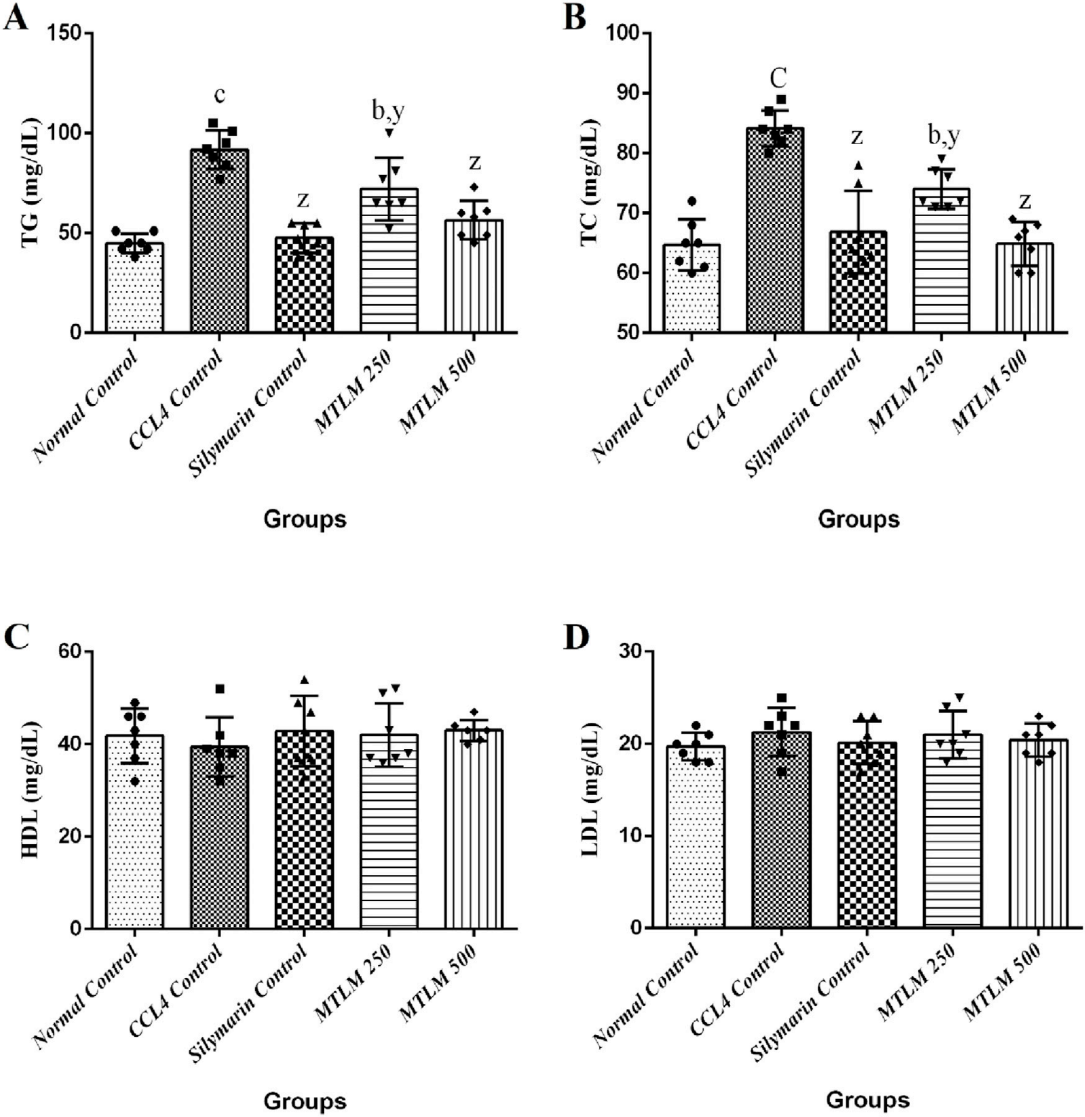
Effects of methanolic *M. thyrsiflora* leaf extract (MTLM) and CCL_4_ on serum lipid profile **(A)** TG, **(B)** TC, **(C)** HDL, and **(D)** LDL. Results, with a sample size of 7, show varying significance: ‘a', ‘b', ‘c' for comparisons with the normal control, and ‘x', ‘y', and ‘z’ with the CCL_4_ control, determined by one-way ANOVA as well as Dunnett’s and Tukey’s *post hoc* test.

The efficacy of *M. thyrsiflora* extracts on lipid metabolism, including serum triglycerides (TG), total cholesterol (TC), high-density lipoprotein (HDL), and low-density lipoprotein (LDL), was tested, and the results are shown in Figure 3. The CCl_4_-induced liver injury group experienced a significant surge in TG levels (*p* ≤ 0.001), indicative of disrupted lipid processing. However, treatment with *M. thyrsiflora*, particularly at higher doses (MTLM 500), showed a significant reduction in TG levels (*p* ≤ 0.001), suggesting a therapeutic effect on lipid metabolism. TC levels also significantly increased in the CCl_4_ group (*p* ≤ 0.001). Still, the extracts, especially MTLM 500, demonstrated a capacity to correct TC levels, aligning closer to the normal control levels (*p* ≤ 0.001) like silymarin. This trend highlights the potential cholesterol-modulating properties of the extract. Regarding HDL, the ‘good’ cholesterol, results indicated that the CCl_4_ group showed a non-significant decline. In contrast, treatment with *M. thyrsiflora*, especially at the higher concentration, resulted in an elevation towards the normal range, though not statistically significant. Lastly, LDL levels, often called ‘bad’ cholesterol, were elevated in the CCl_4_ group but non-significantly. Treatment with both doses of *M. thyrsiflora* extract caused a decrease in LDL levels, though these changes were also not statistically significant. These findings suggest that *M. thyrsiflora* extracts regulate lipid profiles, particularly at a higher dose, potentially benefiting liver health.

### 3.8 Effects on serum biochemical parameters

Total bilirubin (TB) levels serve as a sensitive marker of liver function, reflecting the organ’s ability to process and excrete bilirubin, a byproduct of red blood cell breakdown. Results from this investigation showed that the CCl_4_-treated group exhibited a striking four-fold increase in TB levels compared to the normal control (*p* ≤ 0.001), confirming significant liver damage. However, both doses of the *M. thyrsiflora* leaf extract (MTLM 250 and MTLM 500) effectively mitigated this rise in TB in a dose-dependent manner (*p* ≤ 0.01 and *p* ≤ 0.001, respectively), achieving levels close to those of the normal and silymarin control. Total protein (TP) levels, which encompass a diverse array of proteins synthesized by the liver, also dropped significantly in the CCl_4_-treated group (*p* ≤ 0.001), signifying impaired liver function. Notably, both doses of the *M. thyrsiflora* extract restored TP levels in a dose-dependent manner (*p* ≤ 0.01 for MTLM 250 and *p* ≤ 0.001 for MTLM 500), demonstrating a positive impact on liver protein synthesis like silymarin. Albumin (ALB), a significant component of TP and a marker for nutritional status remained stable across all groups, showing no significant changes. Similarly, globulin (GLB) levels, which include antibodies, were maintained within the normal range upon treatment with *M. thyrsiflora*, CCl_4_, and silymarin. All these findings (Table 3) indicate that *M. thyrsiflora* leaf extracts, particularly at higher doses, can mitigate CCL_4_-induced hepatic impairment, as evidenced by normalized levels of TB, and maintained TP, ALB, and GLB levels.

**TABLE 3:** Effects of methanolic *M. thyrsiflora* leaf extract and CCL_4_ on serum biochemical parameters.

Group	Total protein (g/dL)	Albumin (g/dL)	Globulin (g/dL)	Total bilirubin (mg/dL)
Normal control	6.69 ± 0.37	2.91 ± 0.24	6.05 ± 0.69	0.55 ± 0.05
CCL_4_ Control	5.36 ± 0.47^c^	2.81 ± 0.28	5.78 ± 1.09	2.08 ± 0.07^c^
Silymarin Control	7.01 ± 0.22^z^	3.02 ± 0.18	6.21 ± 0.75	0.65 ± 0.04^b,z^
MTLM 250	6.07 ± 0.13^a,y^	2.95 ± 0.14	5.92 ± 0.67	0.94 ± 0.03^c,z^
MTLM 500	6.91 ± 0.13^z^	2.81 ± 0.14	6.20 ± 0.84	0.70 ± 0.08^c,z^

Results, with a sample size of 7, show varying significance: ‘a', ‘b', ‘c' for comparisons with the normal control, and ‘x', ‘y', and ‘z’ with the CCL_4_ control, determined by one-way ANOVA, as well as Dunnett’s and Tukey’s *post hoc* test.

### 3.9 Effects on anti-oxidative stress markers

**FIGURE 4:**
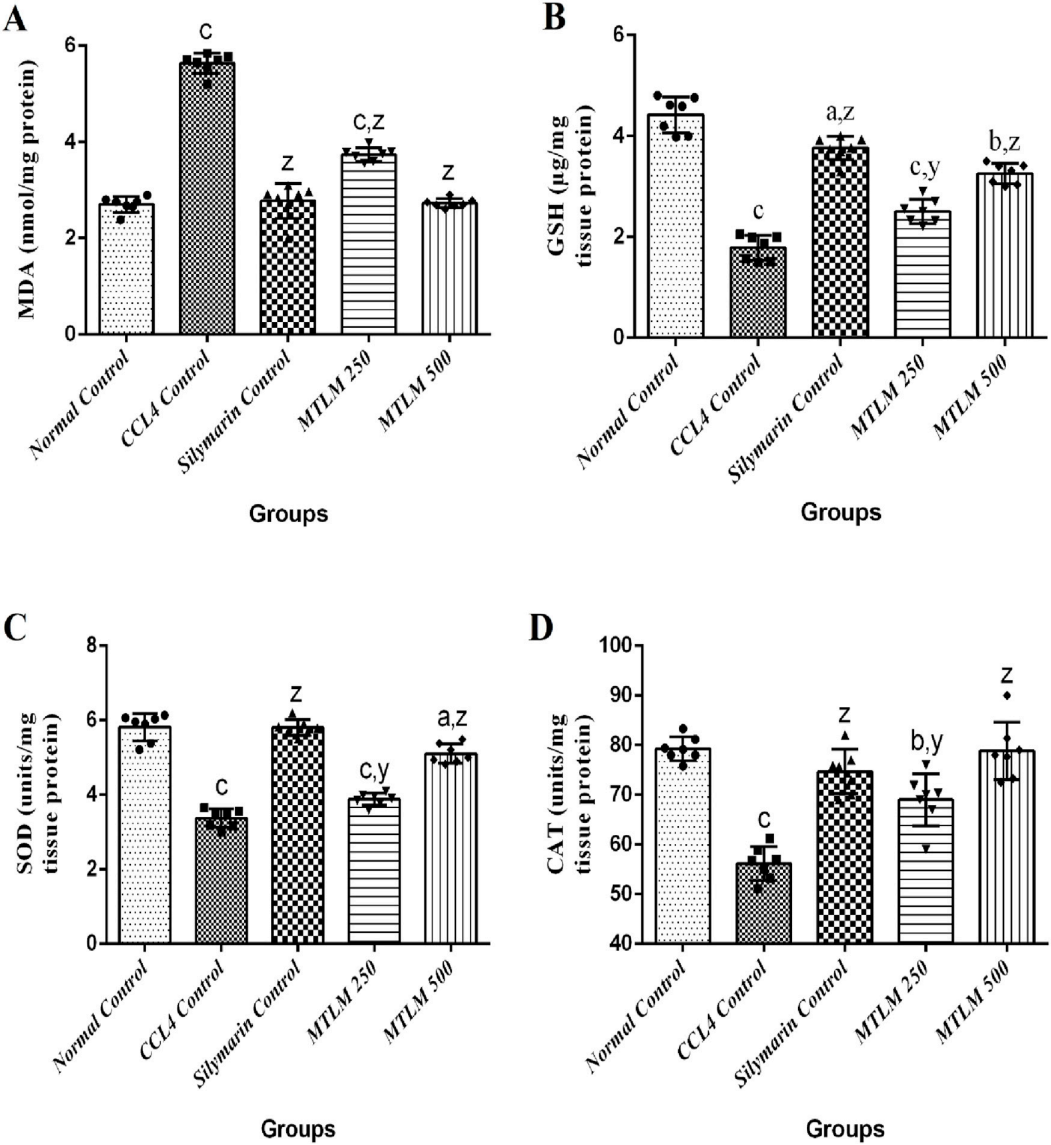
Effects of methanolic *M. thyrsiflora* leaf extract and CCL_4_ on anti-oxidative stress markers. **(A)** MDA, **(B)** SOD, **(C)** CAT. Results, with a sample size of 7, show varying significance: ‘a', ‘b', ‘c' for comparisons with the normal control, and ‘x', ‘y', and ‘z’ with the CCL_4_ control, determined by one-way ANOVA as well as Dunnett’s and Tukey’s *post hoc* test.

In a comprehensive examination of oxidative stress markers following *M. thyrsiflora* treatment, malondialdehyde (MDA) levels, glutathione (GSH) levels, SOD, and CAT activities were evaluated; results are depicted in Figure 4. The MDA levels, a byproduct of lipid peroxidation and an indicator of oxidative stress were significantly higher in the CCl_4_ Control group (*p* ≤ 0.001), suggesting enhanced lipid damage. However, treatment with *M. thyrsiflora*, particularly the 500 mg/kg dosage, significantly reduced MDA levels (*p* ≤ 0.001), indicating a protective effect against oxidative stress. GSH is a major antioxidant that protects liver cells from oxidative damage and maintains cellular redox balance. In this study, levels of GSH were significantly reduced in the CCl_4_ control group (*p* ≤ 0.001), indicating oxidative stress and liver damage. Silymarin and MTLM treatments restored GSH levels, with MTLM 500 showing a notable improvement (*p* ≤ 0.001), though not as high as the Silymarin group. SOD, a critical antioxidant enzyme that mitigates superoxide radicals, was also lower in the CCl_4_ control group (*p* ≤ 0.001), contributing to an oxidative environment. The MTLM treatments increased SOD activities, with the higher dose reaching levels comparable to the normal control (*p* ≤ 0.001), suggesting an enhanced defense against reactive oxygen species. Similarly, CAT, another key enzyme in hydrogen peroxide detoxification, was examined. The CCl_4_ control group’s reduced CAT activity (*p* ≤ 0.001) reflects a compromised antioxidant defense. In contrast, MTLM treatments, especially at higher doses, restored CAT activity to normal levels (*p* ≤ 0.001), suggesting the extract’s antioxidative capacity. These findings highlight the potential of M. thyrsiflora extracts to attenuate oxidative stress by lowering lipid peroxidation and bolstering the antioxidant defense system, as evidenced by the modulation of MDA, SOD, and CAT levels.

### 3.10 Effects on liver histopathology

**FIGURE 5:**
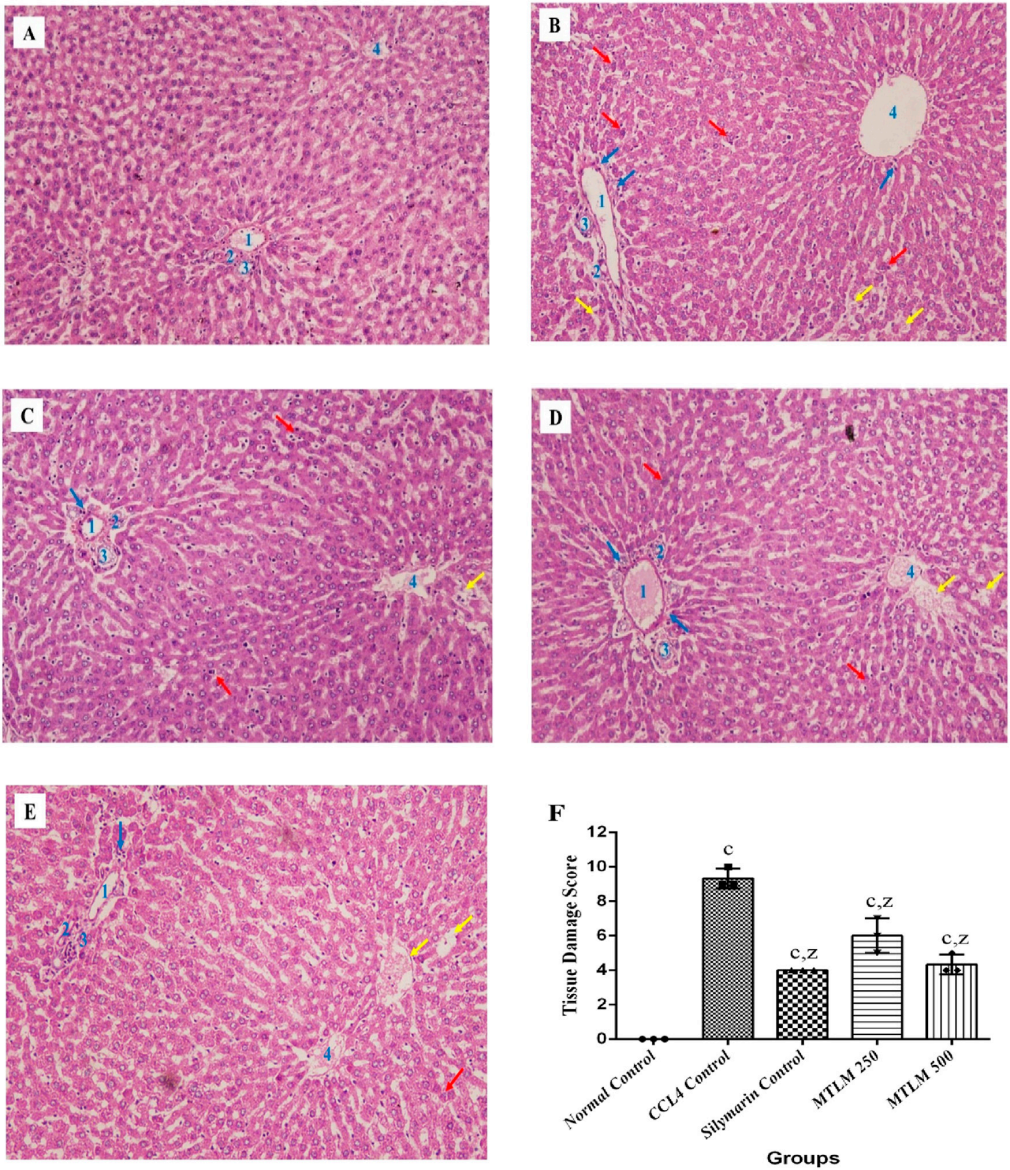
Microscopic views of CCl_4_-induced liver damage and potential protective effects of *M. thyrsiflora*. The normal control **(A)** exhibits organized hepatocytes, while CCl_4_ treatment **(B)** disrupts the architecture, showing necrosis (red arrow), inflammation (blue arrow), and edema (yellow arrow). Silymarin, a known protector **(C)**, minimizes these changes. Notably, both *M. thyrsiflora* extract doses **(D, E)** exhibit significant improvements compared to the CCl_4_-treated group, displaying reduced necrosis, inflammation, and edema, suggesting promising potential for liver protection. **(F)** Histological scoring of hepatic injury. Results were treated in a blinded fashion with three individuals, showing varying significance: ‘a', ‘b', ‘c' for comparisons with the normal control, and ‘x', ‘y', and ‘z’ with the CCL_4_ control, determined by one-way ANOVA as well as Dunnett’s and Tukey’s *post hoc* test. In this Figure, 1 denotes the portal vein, 2 represents the hepatic artery, 3 indicates the bile duct, and 4 signifies the central vein.

The microscopic examination of liver sections revealed that the hepatocytes in the normal control group were well-ordered, radiating from the central vein to the lobule’s edge, with cells displaying standard cytoplasmic consistency, nuclei, and vesicular structures (Figure 5A). In contrast, administration of CCl_4_ resulted in pronounced hepatic disarray, with widespread cell necrosis and noticeable inflammatory infiltration, alongside edema scattered throughout the lobular regions (Figure 5B). On the other hand, treatment with silymarin, used as a reference standard, appeared to mitigate these pathological changes, maintaining hepatocyte integrity with only slight necrotic and edematous manifestations (Figure 5C). Similarly, administration of MTLM extracts in two doses caused a marked histological improvement. Compared to the CCl_4_-induced damage, the treated groups showed only mild to moderate signs of inflammation, hepatocyte degeneration, and edema, indicating the therapeutic potential of *M. thyrsiflora* in liver tissue recovery (Figures 5D, E). Tissue score damage revealed a significant (*p* < 0.001) increment in the CCl_4_ group than the normal control. Administration of both dosses of MTLM extract significantly reduced tissue score damage compared to the CCl_4_ group (*p* < 0.001) (Figure 5F).

## Discussion

Many toxic chemicals such as pesticides, residual food toxins, and environmental pollutants may harm humans, cause serious injury to liver cells, and change the normal hepatic function. In addition, the unwise use of numerous medications can cause hepatic injury and liver disease. In this investigation, the antioxidant capacity and liver-protecting actions of *M. thyrsiflora* leaves against chemically induced liver injury in laboratory animals have been demonstrated for the first time. Our findings showed that *M. thyrsiflora* leaf extracts exert a significant defensive role, mitigating liver damage caused by CCl_4_. In addition, findings from this study highlight the crucial role of antioxidants in combating the harmful effects of free radicals. Excessive production of oxidants disrupts the delicate balance within cells, damaging vital molecules like lipids, DNA, and proteins (Lobo et al., 2010). Fortunately, *M. thyrsiflora* leaves harbor phytochemicals, mainly phenols and flavonoids. These antioxidants shield the cells from free radical attacks by donating electrons or hydrogen atoms, effectively quenching the unpaired electrons. Moreover, they bind metal ions, further hindering the Fenton reaction, a major source of free radical generation (Azat Aziz et al., 2019). Polyphenols even aim at cancer by inhibiting enzymes that activate pro-carcinogens, preventing them from wreaking havoc on DNA (Zhou et al., 2016). Similarly, flavonoids can scavenge free radicals, chelate metal ions, and inhibit free radical-producing enzymes (Kumar and Pandey, 2013).

On the other hand, total antioxidant activity assay, a straightforward method initially used for quantifying vitamin E in seeds, has been expanded to measure the antioxidant potential in plant extracts, including those of *M. thyrsiflora* leaves, which have been shown to possess significant phenolic and flavonoid content, contributing to their overall antioxidant capacity. Within this context, the DPPH assay is a well-established method for determining the antioxidant potential of plant extracts, relying on their capacity for hydrogen donation to neutralize free radicals, as indicated by a change in color (Baliyan et al., 2022). Nitric oxide, despite its role in essential physiological functions like smooth muscle relaxation, neural communication, and immune response modulation, can become harmful when overproduced, leading to various diseases. Its regulatory functions extend to being a neurotransmitter and a vasodilator (Tripathi et al., 2007). In comparative assays, the MTLM extracts demonstrated superior scavenging abilities for DPPH and NO radicals, showing significant activity even when compared to ethyl acetate extract. Similarly, the reducing power of an antioxidant, a measure of its electron-donating ability, reflects its capacity to interrupt lipid peroxidation cascades, and function as both a primary and secondary antioxidant (Vladimir-Knežević et al., 2011) In this regard, the MTLM extracts exhibited commendable reducing capabilities, suggesting their potential as effective antioxidants in biological systems. Findings from this study revealed a direct correlation between the extract’s antioxidant properties and its phenolic content. Thus, the phenolic and flavonoid compounds in *M. thyrsiflora* act as effective electron donors, interrupting the chain reaction of free radicals by transforming them into more stable, less reactive forms.

Hepatic injury, a significant medical issue, arises from various causes and profoundly impacts liver function. The liver, vital for metabolic processes, detoxification, protein synthesis, and hormone regulation, can suffer damage from multiple sources, such as chronic alcohol abuse, long-term use of certain drugs, exposure to environmental toxins, infectious hepatitis viruses, and metabolic disorders. Such damage can lead to an array of liver diseases, including fatty liver, hepatitis, fibrosis, cirrhosis, liver failure, and hepatocellular carcinoma (Satapathy et al., 2015). A central factor in liver injury is oxidative stress, where an imbalance in reactive oxygen species (ROS) production and the body’s detoxification ability leads to cell damage and inflammation. This mechanism is crucial in the progression of various liver diseases. Additionally, altered lipid metabolism resulting in fat accumulation in liver cells (steatosis) is a significant aspect of liver pathology. To understand and effectively treat liver diseases, reliable animal models are essential. In this regard, rodent models are critical in studying human-like liver pathologies such as fibrosis, cirrhosis, and cancer (Delire et al., 2015). These models allow for the exploration of liver functions, responses to harmful agents, and testing potential treatments. CCl_4_-induced hepatic injury is a well-established model for researching liver toxicity because it mimics clinical liver disorders, especially those with oxidative stress, inflammation, and fibrosis, such as non-alcoholic steatohepatitis (NASH) and liver cirrhosis (Rahimi et al., 2022; Wong et al., 2011). This is primarily due to the metabolic activation of CCl_4_ by the cytochrome P450 enzyme system, particularly CYP2E1, which generates trichloromethyl radicals that induce lipid peroxidation and protein oxidation, leading to necrosis and fibrosis (Stoyanovsky and Cederbaum, 1999). In comparison, other models, such as N-nitroso dimethylamine (NDMA/DMN) and N-nitroso diethylamine (NDEA/DEN), are metabolized by cytochrome P450 enzymes to produce alkylating agents that directly damage DNA, leading to mutations and hepatocellular carcinoma (HCC). These models are often employed to study the progression from chronic liver injury to HCC, making them highly relevant for research focused on liver cancer (Da Costa et al., 2014; George et al., 2019). Therefore, the CCl_4_ model is used to study acute liver injury and fibrosis due to its ability to induce oxidative stress and lipid peroxidation (Weber et al., 2003). On the other hand, NDEA is specifically used to induce hepatic carcinogenesis because of its ability to cause DNA alkylation and mutations, leading to the development of liver cancer (Verna et al., 1996). A previous study reported that resveratrol treatment reduces α-fetoprotein levels, improves liver tissue structure, and activates apoptotic markers in NDEA or DEN-induced hepatic carcinoma in rats, suggesting its potential efficacy against hepatocellular carcinoma (Sakthisekaran et al., 2011). Another study revealed that resveratrol prevents NDEA-induced liver cancer in rats by reducing oxidative stress and inflammation, likely through Nrf2 activation, suggesting its potential to prevent human hepatocellular carcinoma (Bishayee et al., 2010). A previous study demonstrated significant molecular differences between NDEA and CCL_4_ of liver injury (Poojari et al., 2010), where embelin, an alkyl benzoquinone derived from *Embelia tsjeriam*-cottam, showed distinct protective mechanisms against NDEA and CCl_4_-induced liver damage. Against NDEA-induced preneoplasia, Embelin exhibited chemopreventive effects by reducing oxidative DNA damage and preventing preneoplastic lesions from progressing to malignant tumors. In contrast, in CCl₄-induced hepatotoxicity, Embelin reduced lipid peroxidation, maintained hepatocyte membrane integrity, and lowered inflammation and necrosis. NDEA treatment led to necrotic changes and abnormal nuclear-cytoplasm ratios, which Embelin treatment significantly reduced. CCl_4_ exposure caused severe pathological changes, including cell swelling and immune cell infiltration. While both agents increased liver enzymes, their effects differed, highlighting their unique mechanisms. Similarly, the root extract of *Operculina turpethum* likely exerts its protective effects against N-Nitrosodimethylamine (NDMA)-induced liver damage through its hepatoprotective and anti-clastogenic effects (Ahmad et al., 2009). Since the primary focus of the research is oxidative stress and lipid peroxidation as they relate to hepatic conditions, the protective effects of the extract against these conditions were evaluated using the CCl_4_ model. Moreover, the CCl_4_ model is advantageous for its consistent and reproducible results, allowing for the study of liver disease at different stages. It is also responsive to preventive and curative treatments, making it an ideal choice for evaluating hepatoprotective agents (Weber et al., 2003).

In this investigation, changes in body weight (BW) and liver weight ratios were employed as crucial indicators of liver toxicity induced by CCl_4_ in rat models. Consistent with previous studies, rats subjected to CCl_4_ exhibited a notable reduction in body weight (Wahid et al., 2016). This weight loss is likely due to the cytotoxic properties of CCl_4_, impacting cellular health. Such effects are in line with prior research demonstrating weight reduction in CCl_4_-treated mice (Sahreen et al., 2011). The liver damage caused by CCl_4_ induces various metabolic disturbances, including diminished appetite, escalated lipid peroxidation, and impaired protein synthesis, collectively contributing to the observed weight loss. Findings from this investigation showed a significant increase in liver size following CCl_4_ administration; this agrees with findings from earlier research (Althnaian et al., 2013). Multiple mechanisms can explain this increase in liver weight. Extended exposure to CCl_4_ typically leads to fibrosis, characterized by the formation of scar tissue within the liver, as identified in previous research (Zhang et al., 2013). Additionally, CCl_4_ has been known to trigger the release of inflammatory mediators, contributing to hepatocyte enlargement and edema, as corroborated by our histopathological observations. Remarkably, treatment with MTLM extracts caused a positive impact on both body and liver weights. This protective effect is likely due to the extract’s antioxidant properties, effectively countering the oxidative stress caused by CCl_4_, thus highlighting *M. thyrsiflora*’s potential in mitigating liver toxicity.

Excessive production of ROS can undermine the effectiveness of crucial antioxidant enzymes like SOD, CAT, and glutathione peroxidase (GPx), compromising their ability to protect cells. This disruption often allows enzymes such as ALT, AST, and ALP to permeate hepatocyte membranes more efficiently, increasing their presence in the bloodstream, a recognized marker of hepatotoxicity. CCl_4_ exacerbates this situation by damaging hepatocytes, disturbing mitochondrial integrity, and enhancing membrane fluidity, as evidenced by elevated levels of these hepatic enzymes in blood tests. This effect has been consistently observed in studies involving CCl_4_ treatment (Chang et al., 2009). Conversely, administration of *M. thyrsiflora* methanol extract in animal models caused a notable protective effect against CCl_4_-induced liver damage, reflected in reduced ALT, ALP, and AST blood levels. Additionally, LDH, another critical enzyme in liver function, usually rises in hepatic impairment, serving as another indicator of liver toxicity (Haque et al., 2018). Our findings revealed that the MTLM extract reduces serum LDH levels, likely due to its phytochemical constituents. GGT is another critical enzyme involved in the liver’s glutathione metabolism and detoxification processes. Elevated GGT levels in the blood are a sensitive marker of liver damage, particularly in conditions affecting the bile ducts or resulting from hepatotoxic substances like CCl_4_ (Koenig and Seneff, 2015). The decrease in GGT levels seen in the groups treated with Silymarin and MTLM indicates a potential preventative or therapeutic impact that might alleviate the liver damage induced by CCl_4_.

The liver’s role in lipid metabolism is crucial, and disruptions in this function can lead to significant alterations in lipid levels. Administration of CCl_4_ causes marked increases in serum TG and TC levels, indicating hepatic impairment (Mahmoodzadeh et al., 2017). This effect can be attributed to reduced protein synthesis and anomalies in phospholipid metabolism, leading to abnormal lipoprotein concentrations. Elevated total cholesterol in the serum might result from increased lipid esterification and diminished oxidation of fatty acids, coupled with slowed lipid clearance. CCl_4_ facilitates the entry of acetate into liver cells, spurring cholesterol synthesis, and enhancing fatty acid and triglyceride production from acetate, while promoting lipid esterification. It also interferes with the release of LDL and the function of lysosomal lipase, potentially contributing to the accumulation of triglycerides in the liver (Vaziri, 2016). Additionally, during CCl_4_ intoxication, lipids from peripheral adipose tissue are known to migrate to vital organs like the liver and kidneys, resulting in lipid accumulation. In contrast, administration of MTLM extracts at a dose of 500 mg/kg reduced serum TC and TG levels, indicating a positive impact on lipid metabolism. This suggests that the extracts from *M. thyrsiflora* might possess properties that counteract the lipid-altering effects of CCl_4_, thereby restoring normal lipid profiles and mitigating liver damage.

Normally, hemoglobin breakdown results in bilirubin production, which the liver excretes via bile. However, severe liver impairment often leads to diminished bilirubin excretion, manifesting as hyperbilirubinemia, an indicator of hepatic damage or necrosis. This phenomenon could be attributed to the compromised ability of the injured liver to effectively process and eliminate bilirubin, which might explain the rise in total serum bilirubin levels following CCl_4_ administration (Fernandez and West, 2005). Interestingly, treatment with the MTLM extract caused a notable prevention of increased bilirubin serum, suggesting its potential to mitigate liver injury. Additionally, the liver plays a crucial role in synthesizing serum proteins. Exposure to CCl_4_ results in a reduction of TP levels, a consequence of impaired protein synthesis, indicating the extent of liver toxicity. In this context, rats treated with CCl_4_ exhibited lowered TP levels, reinforcing the severity of liver damage (Sundari et al., 1997).

However, the administration of *M. thyrsiflora* extract appeared to counteract this effect, as evidenced by elevated serum protein levels, implying a recuperative effect on hepatic cell function. In contrast, our findings showed no significant changes in ALB and GLB levels between the treated and control groups. This stability may be due to the liver’s regenerative and compensatory abilities, which prioritize synthesizing essential proteins like albumin and globulins, even in the face of significant liver injury. The liver may upregulate alternative pathways or allocate resources to maintain these critical serum proteins within normal ranges, despite the overall reduction in total protein levels.

MDA is a harmful byproduct formed during the lipid peroxidation of cell membranes, indicates oxidative stress. Elevated MDA concentrations are symptomatic of excessive free radical activity, leading to oxidative damage in the liver. In cases of CCl_4_-induced liver injury, increased MDA levels reflect enhanced lipid peroxidation, indicating both hepatocellular damage and the failure of the body’s antioxidant defenses to counteract free radical overproduction (Zarezade et al., 2018). This study revealed that treatment of rats with MTLM extract at doses of 250 and 500 mg/kg effectively reduced MDA levels, thereby mitigating lipid peroxidation. This reduction highlights the extract’s potential as a scavenger of free radicals *in vivo*. Corroborating these findings, the *in vitro* experiments utilizing DPPH and NO scavenging assays also demonstrated the extract’s free radical neutralizing ability. On the other hand, GSH is a tripeptide consisting of glutamine, cysteine, and glycine. It plays a vital role as an antioxidant in the body, especially in the liver. GSH is essential for counteracting reactive oxygen species and eliminating toxic compounds, safeguarding cells from oxidative stress and injury. GSH plays a crucial role in the liver by detoxifying xenobiotics, which is essential for preserving hepatic function and avoiding liver damage (Wu et al., 2004). A decrease in GSH levels is a frequent marker of oxidative stress and may cause substantial harm to cells, especially when exposed to hepatotoxic substances such as carbon tetrachloride (Masella et al., 2005). In this study, the marked decrease in GSH levels in the CCl₄ control group highlights the oxidative stress and liver damage induced by CCl_4_, consistent with its known mechanism of generating free radicals that deplete GSH and cause lipid peroxidation in hepatocytes. The Silymarin-treated group exhibits a significant restoration of GSH levels, underscoring Silymarin’s well-documented antioxidant and hepatoprotective properties for the therapy of liver disease (Flora et al., 1998). The MTLM-treated groups also show an increase in GSH levels compared to the CCl_4_ group, with MTLM 500 mg/kg displaying a considerable, though slightly lower, effect than Silymarin. This suggests that MTLM has potential antioxidant effects that help mitigate CCl_4_-induced hepatic damage by replenishing GSH levels and protecting the liver from oxidative stress.

The liver, a critical organ in the human body, functions not only as a major metabolic center but also as an integral part of the body’s endocrine system. It is equipped with robust protective mechanisms, primarily through a range of endogenous antioxidant enzymes like SOD and CAT. These enzymes are crucial in combatting cellular damage caused by free radicals. When exposed to harmful agents like CCl_4_, the liver’s ability to fend off oxidative stress is severely challenged, as evidenced by the decrease in the levels of SOD and CAT. CCl_4_-induced liver injury is largely due to lipid peroxidation, a process where free radicals derived from CCl_4_ cause substantial damage to liver cells (Okamoto et al., 2001). In this respect, antioxidants play a pivotal role in shielding the liver from CCl_4_-induced damage by curbing the production of free radicals. The body’s natural defense systems, including enzymes like CAT and SOD, inhibit and neutralize free radicals, thus preventing liver damage. These enzymes work in synergy to offset the harmful effects of reactive oxygen species (ROS). Mitochondrial dysfunction, often a result of CCl_4_ exposure, disrupts cellular operations, leading to liver damage and cell death. This impairment also skews the balance between ROS production and the antioxidant defense mechanisms. Normally, the liver’s antioxidant system, comprising both enzymatic and non-enzymatic components, effectively neutralizes ROS. SOD plays a unique role in converting excess superoxide radicals into hydrogen peroxide, which is then decomposed by catalase (Weydert and Cullen, 2010). In the context of CCl_4_-induced hepatic injury, there’s a noticeable reduction in the activity of these vital enzymes. However, our findings revealed that the administration of MTLM extracts resulted in a marked elevation in the activities of SOD and CAT in treated animals. This indicates the extract’s significant potential in enhancing the liver’s antioxidant defense system, thereby providing effective protection against oxidative stress and liver damage typically associated with exposure to CCl_4_.

**FIGURE 6:**
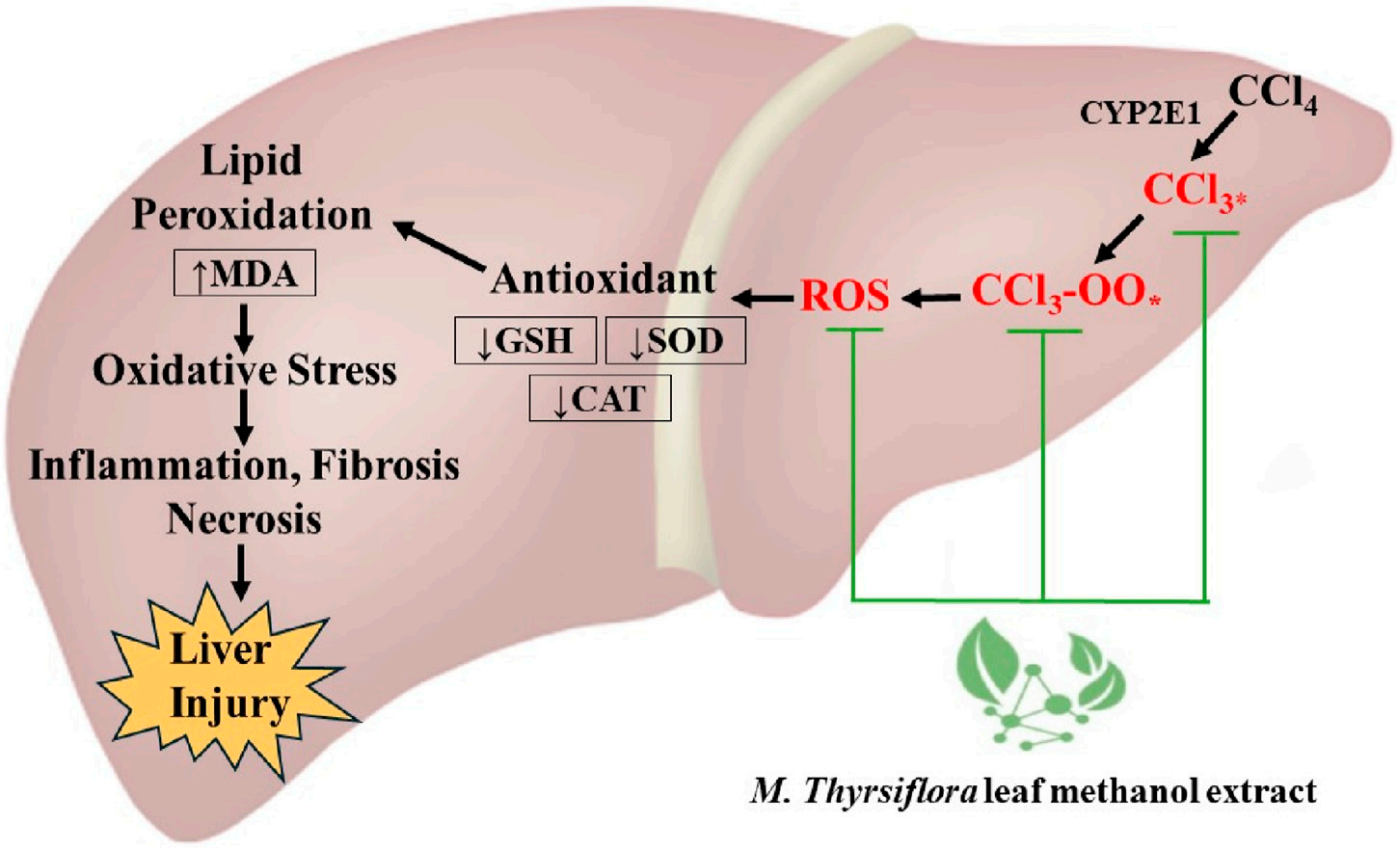
Schematic representation of the hepatoprotective mechanism of *M. thyrsiflora* leaf methanol extract against CCl₄-induced liver injury.

Histopathological analysis is critical in evaluating liver cell structural abnormalities (Yoshioka et al., 2018). In this study, liver samples from rats solely treated with CCl_4_ displayed typical signs of liver toxicity, including hepatocyte deterioration, necrosis, inflammation, and edema-induced vascular congestion. These pathological changes are characteristic of CCl_4_-induced hepatocyte damage in rats (Scholten et al., 2015). In contrast, histological examinations of rats administered with MTLM extracts and silymarin showed notable cellular preservation. This was evident from the diminished incidence of necrosis, reduced infiltration of inflammatory cells, and lessened edema in blood vessels. The protective effects of *M. thyrsiflora* on liver cells align with the biochemical observations, where an improvement in liver function was noted. The efficacy of the *M. thyrsiflora* extract in combating hepatic damage caused by CCl_4_ is apparent from its ability to correct cellular irregularities and enhance the overall architecture of liver tissue. This protective effect is further supported by the increased activities of intrinsic antioxidant enzymes and the decrease in lipid peroxidation levels observed in the extract-treated group. Thus, MTLM extracts demonstrate a potent hepatoprotective effect, evident both at the biochemical and histopathological levels, indicating its potential therapeutic value in liver damage recovery. Taken all together, due to its richness in phenolic and flavonoid compounds, the *M. thyrsiflora* leaf extract reduces oxidative stress by neutralizing reactive oxygen species, as reflected in the CCl_4_-induced liver damage model, where it enhances antioxidant enzyme activities and lowers lipid peroxidation, proving it would be a promising natural liver protectant. Figure 6 depicts an overall plan of potential hepatoprotective effects of *M. thyrsiflora*. Generally, CCl_4_ is metabolized by the cytochrome P450 enzyme CYP2E1, generating trichloromethyl radicals (CCl_3_*) and trichloromethyl peroxy radicals (CCl_3_-OO*). These ROS cause lipid peroxidation, leading to an increase in MDA levels, oxidative stress, and subsequent inflammation, fibrosis, and necrosis, resulting in liver injury. *M. thyrsiflora* leaf extract reduces oxidative stress by neutralizing reactive oxygen species, as evident by increased amounts of antioxidant enzyme activity and lower lipid peroxidation.

This study presents several strengths, notably its comprehensive evaluation of antioxidant and hepatoprotective properties. A well-established CCl_4_-induced liver injury model was used, demonstrating the extract’s potential in reducing oxidative stress and enhancing antioxidant enzyme activities *in vivo*. The results, supported by both biochemical and histopathological analyses, underscore the efficacy of MTLM in mitigating liver damage. However, the study has a few limitations. While the extract showed promising antioxidant and hepatoprotective effects, there was no detailed characterization of the plant’s active compounds. Identifying specific constituents would provide greater insight into the mechanisms of action and potential therapeutic uses. Additionally, the study did not incorporate molecular markers to understand the pathways involved in the hepatoprotective effects. This omission limits the understanding of the specific molecular interactions and protective mechanisms. Furthermore, while the removal of most of the solvent was ensured using rotary evaporation under reduced pressure, it is acknowledged that trace amounts of ethanol may still be present in the dried extracts. This could be considered a limitation of our study, as the absence of a dedicated solvent control might not entirely rule out the possibility of minimal solvent effects on the observed outcomes. Future studies should focus on isolating and characterizing the active compounds in *M. thyrsiflora* and utilize molecular techniques to identify key signaling pathways. Additionally, a dedicated solvent control should be included to fully account for any potential effects of residual solvent on the results Such research would enhance the understanding of the therapeutic potential of *M. thyrsiflora* and its application for liver disease management.

In summary, findings from this study highlighted the potent antioxidant and hepatoprotective effects of MTLM against CCl_4_-induced liver damage in rats. The extract, rich in phenolic and flavonoid compounds, effectively neutralized harmful free radicals, protecting against oxidative liver injury. The CCl_4_ model demonstrated the extract’s capacity to enhance antioxidant enzyme activities and reduce lipid peroxidation, supported by histopathological and biochemical evidence. These findings support the traditional medicinal use of *M. thyrsiflora*, suggesting it is a promising natural therapeutic agent against liver toxicity and oxidative stress. Further studies are needed to establish its molecular mechanisms, safety profiling, and active constituents’ characterization.

## Data Availability

The original contributions presented in the study are included in the article/supplementary material, further inquiries can be directed to the corresponding authors.
